# Hydroxyapatite Surfaces Functionalized with a Self-Assembling Peptide: XPS, RAIRS and NEXAFS Study

**DOI:** 10.3390/nano10061151

**Published:** 2020-06-12

**Authors:** Valeria Secchi, Stefano Franchi, Monica Dettin, Annj Zamuner, Klára Beranová, Alina Vladescu, Chiara Battocchio, Valerio Graziani, Luca Tortora, Giovanna Iucci

**Affiliations:** 1 Department of Science, Roma Tre University, Via della Vasca Navale 79, 00146 Rome, Italy; valeria.secchi@uniroma3.it (V.S.); chiara.battocchio@uniroma3.it (C.B.); luca.tortora@uniroma3.it (L.T.); 2 Department of Materials Science, University of Milano-Bicocca, Via Cozzi 55, 20125 Milan, Italy; 3 Institute of Structure of Matter (ISM), National Research Council (CNR), Via Fosso del Cavaliere 100, 00133 Rome, Italy; 4 Department of Industrial Engineering, University of Padua, Via Marzolo 9, 35131 Padua, Italy; monica.dettin@unipd.it (M.D.); annj.zamuner@unipd.it (A.Z.); 5 Materials Science Beamline, Elettra Sincrotrone Trieste SCpA, Strada Statale 14, km 163.5, 34149 Basovizza-Trieste, Italy; klara.beranova@fzu.cz; 6 Department for Advanced Surface Processing and Analysis by Vacuum Technologies, National Institute of Research and Development for Optoelectronics, 409 Atomistilor St., 077125 Magurele, Romania; alinava@inoe.ro; 7 Physical Materials Science and Composite Materials Centre, National Research Tomsk Polytechnic University, Lenin Avenue 43, 634050 Tomsk, Russia; 8 Surface Analysis Laboratory, INFN University Roma Tre, via della Vasca Navale 84, 00146 Rome, Italy; valerio.graziani@roma3.infn.it

**Keywords:** hydroxyapatite, self-assembling peptides, magnetron sputtering, Mg, Si and Ti dopants, XPS, NEXAFS, FTIR

## Abstract

Hydroxyapatite (HAP) coatings can improve the biocompatibility and bioactivity of titanium alloys, such as Ti6Al4V, commonly used as material for orthopedic prostheses. In this framework, we have studied the surface of HAP coatings enriched with Mg and either Si or Ti deposited by RF magnetron sputtering on Ti6Al4V. HAP coatings have been furtherly functionalized by adsorption of a self-assembling peptide (SAP) on the HAP surface, with the aim of increasing the material bioactivity. The selected SAP (peptide sequence AbuEAbuEAbuKAbuKAbuEAbuEAbuKAbuK) is a self-complementary oligopeptide able to generate extended ordered structures by self-assembling in watery solutions. Samples were prepared by incubation of the HAP coatings in SAP solutions and subsequently analyzed by X-ray Photoelectron Spectroscopy (XPS), Fourier Transform Infrared (FTIR) and Near Edge X-ray Absorption Fine Structure (NEXAFS) spectroscopies, in order to determine the amount of adsorbed peptide, the peptide stability and the structure of the peptide overlayer on the HAP coatings as a function of the HAP substrate and of the pH of the mother SAP solution. Experimental data yielded evidence of SAP adsorption on the HAP surface, and peptide overlayers showed ordered structure and molecular orientation. The thickness of the SAP overlayer depends on the composition of the HAP coating.

## 1. Introduction

Titanium and its alloys are renowned biocompatible materials that are commonly used for dental and orthopedic prostheses. Ti6Al4V, for instance, is typically employed in clinical practice as a biocompatible material in the manufacturing of hip and artificial knee joints and dental implant prostheses components [1,2,3,4,5]. Improved biocompatibility and bioactivity of the implant can be obtained by appropriate modifications of the alloy surface by bioactive coatings.

For instance, hydroxyapatite (HAP) coatings show good bioactive ability and are known to support bone growth at the interface between the implant and the extracellular matrix [6,7]; therefore, HAPs have been used to coat orthopedic and dental implants [8,9,10].

In previous papers, some of us reported the preparation of HAP coatings, enriched with Mg and either Si or Ti deposited on the Ti6Al4V alloy by RF magnetron sputtering [11,12]. The HAPs incorporating different types of ions were investigated in order to achieve better control over osteoblast adhesion to HAP coatings. For instance, magnesium (Mg^2+^) or other doubly charged cations can replace calcium (Ca^2+^) in the biological apatite lattice, mimicking the complex chemistry of the human bone [13], stimulating osteogenesis [14]. Titanium was chosen due to its biocompatibility and ability to promote cell growth [15].

Silicon, on the other hand, in contact with simulated body fluids, can form silanol groups (Si–OH) that support the migration and deposition of calcium and phosphate ions, promoting the growth of a bone-like apatite layer and easing osteoblast attachment [16]. 

Functionalization of the material surface with bioactive molecules (for instance, osteogenic growth factors or cell adhesion sequences), that can be immobilized on the metal surface and establish a chemical interaction with host’s cells [4,17], is a possible pathway to increase the biocompatibility of orthopedic titanium-based implants and promote osseointegration. 

Adsorption or immobilization of peptides on the HAP surface was recently investigated by several authors [18,19] who studied the influence of the peptide-HAP interactions on peptide chain folding [18] or the immobilization of adhesion peptides with the aim of stimulating bone regeneration [19,20]. In particular, Durrieu et al. studied the covalent immobilization of RGD-peptides on HAP in order to promote osteoblast adhesion [20]. Peptide sequences rich in aspartate and glutamate are known to bind to HAP surfaces [21], through interaction of the carboxylate anions in the peptide pending groups with the Ca^2+^ cations of HAP.

In this work, we present a spectroscopic study on the adsorption of the self-assembling peptide EAbuK 16-II (simply indicated as SAP in the following text) on the HAP coatings surfaces containing Mg, Si and Ti. EAbuK 16-II shows a self-complementary sequence that consists of a regular alternation of apolar and charged amino acids and of positive and negative charges (complete peptide sequence H-Abu-Glu-Abu-Glu-Abu-Lys-Abu-Lys-Abu-Glu-Abu-Glu-Abu-Lys-Abu-Lys-NH_2_, where Abu = α-aminobutyric acid, hydrophobic residue; E = Glu = glutamate, negatively charged residue, K = Lys = lysine positively charged residue); this sequence induces the formation of extended ordered structures by self-assembling from water solutions. Self-assembling peptides (SAPs) are a promising class of synthetic materials, since they can self-organize into nanostructures from aqueous solution and adhere to the surface of biocompatible material as a scaffold coating [22,23]. SAPs scaffolds form a biomimetic matrix that can support the growth of selected cells, e.g., osteoblast, by imitating the extracellular matrix structure [4].

HAP-coated Ti6Al4V samples were incubated in SAP solution and analyzed before and after incubation, by surface-sensitive techniques such as XPS (X-ray Photoelectron Spectroscopy), FTIR (Fourier Transform Infrared Spectroscopy), both in RAIRS (Reflection–Absorption Infrared Spectroscopy) mode and by FTIR microspectroscopy, and NEXAFS (Near Edge X-ray Absorption Fine Structure) spectroscopy.

## 2. Materials and Methods

### 2.1. Sample Preparation

HAP coatings were prepared by RF magnetron sputtering technique on Ti6Al4V substrates (Bibus Metal Ag), using three cathodes made of HAP, MgO and either TiO_2_ or SiC. Because we intended to obtain coatings with small amounts of dopants, we used the cathodes made of oxides (MgO and TiO_2_) or carbide (SiC). These cathodes were selected due to the similar deposition rate with that of HAP established after preliminary investigations. Before the depositions, substrates were pretreated as described in References [11,12]. Deposition conditions of the analyzed samples were base pressure = 1.3 × 10^−4^ Pa; Ar pressure = 6.6 × 10^−1^ Pa; bias voltage = −60 V; and deposition temperature = 700 °C. RF power fed applied on each used cathode is listed in Table 1. 

EAbuK16-II (SAP in the following) was synthesized as described in Reference [24]; sample purity of 95% was determined by RP-HPLC (Waters mod. 1525) chromatogram integration, identity confirmed by ESI-ToF (Waters Xevo G2-S QTof) analysis.

Substrates were incubated for 18 h in aqueous solution containing 1 mg/mL of SAP dissolved in 10 mM NaCl, at two different pH conditions: 0.100 mM HCl (J. T. Baker c/o Fisher Scientific Italia, Rodano (MI), Italy) (“pH 4”) or 0.100 mM NaOH (Carlo Erba, Milano, Italy) (“pH 10”). 

After incubation, all samples were washed thrice with NaCl 0.10 M at pH 7 and thrice with distilled water, in order to remove all the SAP molecules weakly bonded to the HAP surface.

### 2.2. Samples Investigation

#### 2.2.1. X-ray Photoelectron Spectroscopy (XPS)

XPS analyses were performed in a homemade instrument consisting of two chambers (preparation and analysis) separated by a gate valve. The analysis chamber is equipped with a manipulator having six degrees of freedom and with a 150 mm mean radius hemispherical electron analyzer with a five-lens output system (operating at a pass energy of 25 eV, during the experiments) combined with a 16-channel detector (Multiplier Voltage used 1950 eV). Measurements were performed at normal take-off angle (θ = 90°). The analyzed surface area has a diameter of ~100 μm. Samples were introduced in the preparation chamber, left outgassing overnight, at a pressure of about 10^−8^ Torr, and subsequently introduced in the analysis chamber. Vacuum in the analysis chamber during measurements was in the 10^−9^–10^−10^ Torr range. Mg Kα non-monochromatized X-radiation (hν = 1253.6 eV) was used to record Ca2p, P2p, Ti2p, Si2p, Mg2p, O1s, C1s and N1s photoemission spectra on the respective samples. All samples were analyzed twice, in order to check data reproducibility.

All spectra were energy referenced to the Ca2p3/2 of hydroxyapatite, used as internal standard and located at a Binding Energy BE = 347.5 eV, as reported in [25,26]; the accuracy of this calibration was checked by comparing the BE values measured on the respective samples for the Ti2p signal of TiO_2_ and for the N1s and C1s signals of the peptides with reported data [22,23]. Alignment to the C1s peak of adventitious carbon was avoided, since recent publications showed that this method is not always reliable [27,28]. The experimental spectra were subsequently analyzed via a curve-fitting procedure, using Gaussian curves having full width at half maximum FWHM = 1.7–2.2 eV as fitting functions. Atomic ratios were calculated from peak intensities, using Scofield’s cross-section values as normalization factors.

#### 2.2.2. Reflection–Absorption Infrared Spectroscopy (RAIRS)

RAIRS measurements in the wavenumber range 400–4000 cm^−1^ were carried out with a VECTOR 22 (Bruker, USA) FTIR interferometer (resolution 1 cm^−1^), equipped with a Specac P/N GS19650 series monolayer/grazing angle accessory and with a DTGS detector. Spectra were recorded at 70° incidence angle of the impinging radiation relative to the normal to the sample surface. 

#### 2.2.3. Fourier Transform Spectroscopic Imaging (μFTIR)

An FTIR microscope (Nicolet iN10 MX, Thermo Scientific, USA) with liquid nitrogen cooled MCT (mercury-cadmium telluride) detector was employed for acquiring FTIR spectra and maps of the HAP coatings. Spectra were collected in reflection mode in the region of 400–4000 cm^−1^ (resolution 4 cm^−1^). In particular, for FTIR mapping, the rectangular aperture was set at 300 × 300 m^2^.

#### 2.2.4. Near Edge X-ray Absorption Fine Structure (NEXAFS) Spectroscopy

Near Edge X-ray Absorption Fine Structure (NEXAFS) spectroscopy experiments were performed at the Material Science beamline (MSB) of the Elettra synchrotron (Trieste, Italy). MSB, placed at the left end of the bending magnet 6.1, is equipped with a plane grating monochromator that provides estimated 80–90% linear polarized light in the energy range of 21–1000 eV. The UHV end station, with a base pressure of 1 × 10^−10^ torr, is equipped with a Specs Phoibos 150 hemispherical electron analyzer.

NEXAFS spectra were acquired at the C and N K-edges, using the carbon and nitrogen KVV Auger yields, at normal (90°), grazing (20°) and magic angle (54.7°) incidence of the photon beam, with respect to the sample surface. Energy resolution for the C and N K-edge NEXAFS spectra is estimated to be 0.23 and 0.38 eV, respectively. 

The raw NEXAFS spectra have been normalized to the intensity of the photon beam. Then, the corresponding background spectra of the clean samples acquired under identical conditions were subtracted.

Spectra were then normalized by subtracting a straight line that fits the part of the spectrum below the edge and assessing to 1 the value at 320.00 and 425.00 eV for carbon and nitrogen, respectively.

## 3. Results

### 3.1. Analysis of Pristine Samples

#### 3.1.1. XPS Investigations

**Figure 1:**
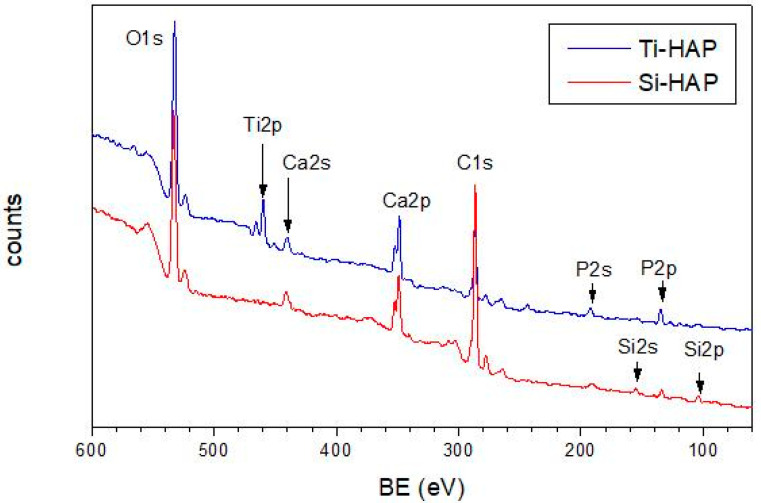
Wide-scan XPS spectra of the pristine Ti-HAP and Si-HAP samples in the 60–600 eV range.

Prior to incubation in SAP solution, the pristine Ti-HAP and Si-HAP samples indicated in Table 1 were investigated by XPS. Wide-scan surveys along with assignment of the main photoemission signals are shown in Figure 1.

The single photoemission peaks were subsequently measured in more detail and analyzed. Their binding energies (BEs) are presented in Table 2. In the case of phosphorus, silicon and magnesium, the two 2p spin–orbit components cannot be resolved in the reported experimental conditions, and, therefore, we present BEs of the maximums of these spectral structures.

For sample Ti-HAP, the measured BE of P2p signal is in good agreement with the value reported for hydroxyapatite [25,26,29]. Furthermore, the Ti2p_3/2_ peak is located at a BE typical of TiO_2_, and the measured BE of Mg2p is in agreement with the expected value for MgO [30,31,32]. 

The O1s signal results from two components; the main peak (1) is located at 531.3 eV and corresponds to hydroxyapatite [25,26,29]. A low BE shoulder at about 530.4 eV is related to the negatively charged oxygens originating from TiO_2_ and MgO [30,31,32].

For sample Si-HAP, the P2p signal appears at slightly lower BE compared to sample Ti-HAP, but can be still ascribed to phosphate anions. The measured BE for Si2p signal is not consistent with the values expected for SiC (~100.5 eV [30]) and reveals a substantial oxidation; while a BE of about 103.3 eV is expected for silica, silicates show BEs in the 102.5–103.0 eV range, which is in agreement with the measured value of 102.6 eV [32]. The O1s spectrum shows only one broad component; the measured binding energy is intermediate between the values expected of phosphates and silicates. Therefore, the spectral feature can be considered as a combination of two indistinguishable peaks corresponding to oxygen originating from phosphate and silicate ion groups [30]. The measured intensity for signal Mg2p is very low; this result is not unexpected, considered the low Mg content (1.9 ± 0.02 at. %) estimated for this sample by EDS measurements [12]. Accordingly, no low BE component related to MgO could be detected in the O1s spectrum.

The investigated samples also show a C1s peak located at approximately 285.0 eV BE that can be related to aliphatic carbons due to surface contamination that cannot be completely removed in the reported experimental conditions. Measured atomic ratios are shown in Table 3. 

The expected P/Ca ratio for HAP (chemical formula Ca_5_(PO_4_)_3_(OH)) is 0.6; the measured atomic ratio is in good agreement with the expected value for sample Ti-HAP, but lower than expected for the Si-HAP sample. The measured Si/Ca ratio for the Si-HAP sample shows that part of the Ca^2+^ cations are neutralized by silicate anions. 

The measured magnesium content is higher for Ti-HAP than for Si-HAP. Finally, only for the Ti-HAP sample, the low BE oxygen species detected (O_0_), related to TiO_2_ and MgO, are present in a fairly high atomic ratio with P-O oxygens (O_1_). For Si-HAP, having a lower Mg content and obviously no titanium, the intensity of signal O_0_ is too low to be detected.

#### 3.1.2. FTIR Investigations

**Figure 2:**
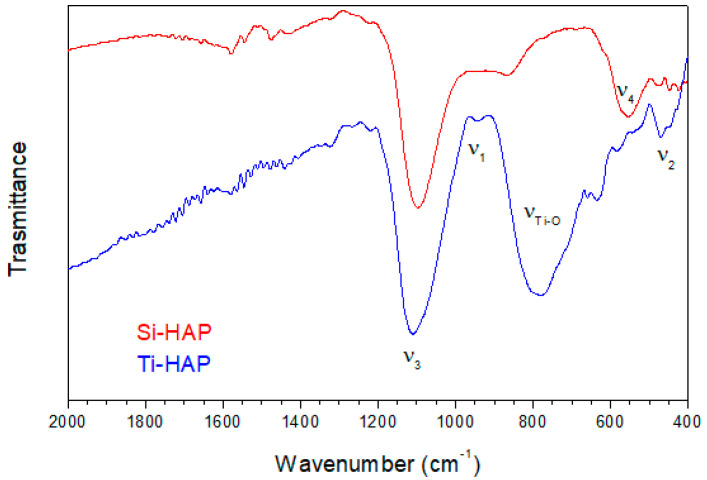
FTIR spectra of the pristine HAP samples.

The infrared spectra of the investigated samples in the range 2000–400 cm^−1^ are shown in Figure 2.

According to the literature, in the IR spectrum of hydroxyapatite, the PO43- anion shows four peaks, labeled ν_1_−ν_4_ and corresponding respectively to symmetric stretching mode (ν_1_, 938 cm^−1^), doubly degenerate bending mode (ν_2_, 420 cm^−1^), triply degenerate antisymmetric stretching mode (ν_3_, 1017 cm^−1^) and to triply degenerate bending mode (ν_4_, 567 cm^−1^) [33]. The antisymmetric stretching mode ν_3_ yields a very intense absorption band; the exact peak position can change in mixed apatite systems, with the ν_3_ band resulting from three component peaks located at 1096, 1085 and 1056 cm^−1^ [34].

In the spectra of all the analyzed samples, the ν_3_ band is clearly evident and located at 1080–1090 cm^−1^, and a weak band ν_1_ is also present at about 940 cm^−1^. The Si-HAP sample shows an intense ν_4_ band located at 560 cm^−1^; for the Ti-HAP sample, the peak is less intense and shifted to 580 cm^−1^. Finally, both samples show a weak peak ν_2_ at about 450–460 cm^−1^.

The strong and broad band at about 800 cm^−1^ in the spectrum of Ti-HAP is related to Ti-O and Ti-O-Ti stretching vibrations of TiO_2_ [35]. For the Si-HAP sample, the silicon carbide deposited by magnetron sputtering appears oxidized to silicate from XPS results. The most intense peak in the IR spectra of silicates is related to the stretching vibration of SiO44- tetrahedra (ν_3_), with components at 938, 906 and 883 cm^−1^ [36]. This peak can be detected in the spectra of both samples as a shoulder on the low wavenumber side of the main ν_3_ band due to PO43- anions.

### 3.2. SAP Adsorption on the HAP Surface

#### 3.2.1. FTIR and XPS Results

HAP samples were incubated in SAP solutions with pH values of 4 and 10 pH, as described in the experimental section. Previous investigations on SAP adsorption on the TiO_2_ surface showed that the thickness of the peptide overlayer decreases with increasing pH of the mother solution [23,37]; however, in the 4–10 pH range, the thickness of the peptide overlayer remains approximately constant, and only at pH = 12 a significant decrease of the overlayer thickness can be observed [23]. On the other hand, the solubility of hydroxyapatite is expected to increase as the solution’s pH decreases. 

A survey of the FTIR spectra of the investigated samples in the 1400–400 cm^−1^ range, which comprises the main diagnostic bands of HAP, after incubation of the HAP samples in SAP solutions, showed no changes at the investigated pH values, thus offering of the stability of the HAP coating, even in a mildly acidic environment. 

XPS investigations yield evidence of peptide adsorption on the HAP surface, as the measured atomic ratios measured after incubation of the HAP samples with the SAP solutions, as shown in Table 4, prove. 

Compared to the pristine samples, the P/Ca atomic ratio shows an evident increase, probably due to partial HAP solubilization on the outmost surface, and/or to rearrangement of the HAP surface, possibly with migration of phosphate anions from the bulk to the surface. An increase is also detected in the Ti/Ca or respectively Si/Ca ratios at high pH values. Moreover, the Mg2p signal, already very low in pristine coatings, is now not observable, due to the SAP coverage. However, the absence of modification of the bands related to HAP in the FTIR spectra evidence a substantial stability of the HAP coating. The apparent contradiction between XPS and FTIR results is related to the different sampling depth of the two techniques. Our comparison between XPS and FTIR results suggests that the modifications in the HAP structure due to incubation with SAP solutions involve only the first 50 Å of the coating surface, while at higher sampling depth, the HAP structure is unperturbed.

The main evidence of the SAP adsorption on the HAP surface is the appearance of a new N1s signal, related to the peptide nitrogens. At the same time, changes are evidenced in the C1s and O1s spectra, and a strong increase in the C/Ca and O/Ca ratios is measured.

**Figure 3:**
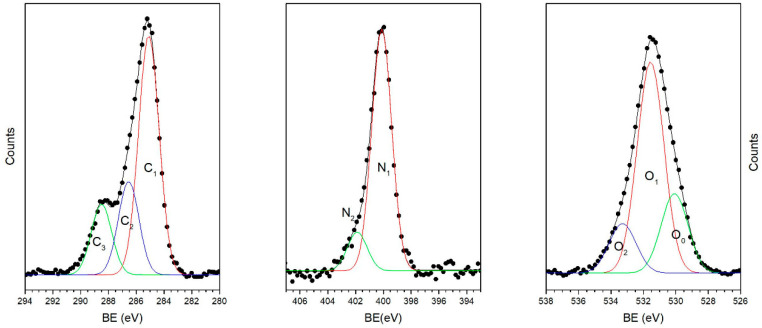
The C1s, N1s and O1s spectra of sample Ti-HAP after SAP adsorption at pH 4 and curve-fitting analysis. Markers represent experimental points, lines calculated spectra and fitting components.

The C1s, N1s and O1s spectra of sample Ti-HAP at pH = 4 are shown in Figure 3.

The C1s signal shows a complex structure and can be decomposed by curve fitting into three component peaks labeled C_1_–C_3_ in Figure 3. Peak C_1_, located at BE = 285.0 eV, corresponds partially to aliphatic C–C carbons of the amino acids side chains and partially to surface contamination; peak C_2_, located at 286.4 eV is due to C–N carbons of the main peptide chain and of the lysine side chains; peak C_3_, located at 288.4 eV, is related to the N–C=O peptide bond carbons of the SAP backbone and to O–C=O carbons of the glutamate side chains [31]. The measured atomic ratios between the three components are shown in Table 4. The calculated ratios between the different carbon atoms in the SAP EAbuK16-II is C_1_:C_2_:C_3_ = 1:0.55:0.55.

In the N1s spectrum, the main signal (N_1_) at 400.1 eV BE is related to the unprotonated nitrogens of the peptide backbone, and the higher BE component (N_2_, BE = 401.9 eV) is due to the protonated nitrogens of the lysine pending groups, that pair by ionic bond with the carboxylate groups of glutamate in the self-assembled structure, a structure that is known to be stable in a wide pH range [23]. In the SAP, the ratio between lysine and peptide backbone nitrogens is 0.2, and the measured N_2_/N_1_ ratios (Table 4) are in fairly good agreement with the expected value. For samples showing a very low N/Ca ratio, the intensity of the N1s signal is too low to allow decomposition of the overall signal in the two component peaks, N_1_ and N_2_. 

It is worth noticing that, according to Table 4, the samples with a high C/Ca ratio also show a high N/Ca ratio and higher C_2_/C_1_ and C_3_/C_1_ ratios.

The O1s spectrum results from three components, labeled O_0_-O_2_ in Figure 3. The first two peaks were already present in the pristine sample spectra, located approximately at the same BE, and were attributed, respectively, to TiO_2_ oxygens (O_0_ BE = 530.2 eV) and to phosphate oxygens of hydroxyapatite (O_1_ BE = 531.6 eV). After peptide adsorption, the O_0_/O_1_ atomic ratio decreases, since contributions to peak O_1_ can also be due to the oxygens of the peptide backbone, (N–C=O*) located approximately at the same BE. A third peak (O_2_) appears BE = 533.3 eV; a similar signal has been evidenced in the O1s spectra of SAP deposited on titanium and attributed to C–O carbons and physisorbed water [22,23].

The N/Ca and C/Ca ratios shown in Table 4 appear significantly higher for sample Ti-HAP compared to sample Si-HAP, at both pH values, suggesting that TiO_2_ actually plays a role in supporting peptide adsorption. Moreover, for Ti-HAP, the amount of immobilized peptide is higher at low pH, while for Si-HAP, apparently there is no clear influence of the pH of the mother solution.

The thicknesses of the peptide overlayer on the HAP surfaces, shown in the last column of Table 4 (Å), was calculated from the attenuation of the Ca2p_3/2_ signal due to peptide immobilization, according to the following Equation (1):

I = I_0_exp(−d/λ) (1)

where I and I_0_ are the Ca2p_3/2_ signal intensities before and after SAP immobilization, d is the peptide overlayer thickness, and λ is the inelastic mean free path calculated according to the following Equation (2):

λ = B (KE)^1/2^ (2)

where KE is the photoelectron kinetic energy, and B = 0.087 nm (eV)^−1/2^ for organic materials [38].

The thickness of the organic overlayer adsorbed on the surface of Ti-HAP is evidently higher than the one on Si-HAP, suggesting the hypothesis that TiO_2_ incorporated in the HAP lattice eases peptide immobilization.

The presence and the structure of the SAP adsorbed on the HAP coatings was also investigated by FTIR microscopy.

**Figure 4:**
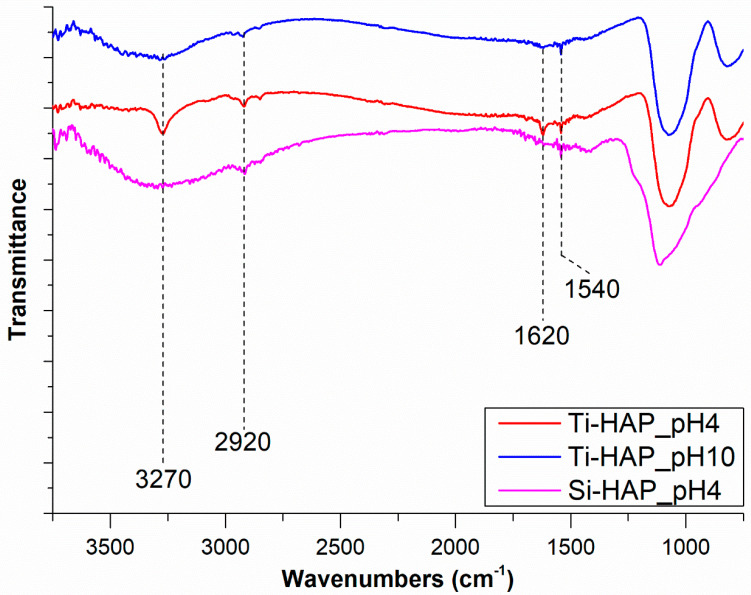
FTIR spectra in the 4000–800 cm^−1^ region of samples Ti-HAP and Si-HAP, after incubation in SAP solutions at different pH values.

The spectra of sample Ti-HAP after incubation with SAP solution at pH = 4 and 10 and of sample Si-HAP after incubation at pH = 4, shown in Figure 4, reveal the presence of new SAP-related bands. In particular, the peak at 3270 cm^−1^ is due to N–H stretching vibrations of the peptide bonds, the peak at 2920 cm^−1^ to C–H stretching of the pending groups and the peaks at 1620 cm^−1^ and 1540 cm^−1^ can be attributed to C=O stretching vibrations (amide I band) and to N–H bending (amide II), respectively. In the IR spectra of proteins and peptides, the position of the amide (I) band can be used to determine the peptide secondary structure [39,40]. In particular, the amide (I) is found around 1650 cm^−1^ in peptides with α-helix or random coil conformation and located between 1620 and 1640 cm^−1^ in β-sheets, appearing at about 1635 cm^−1^ for parallel and at 1615–1625 cm^−1^ for antiparallel β-sheets; a stronger interchain hydrogen bond results in a lower frequency of the amide (I) band. In previous investigations on the FTIR spectra of EAbuK16-II, the amide (I) band was always found at 1620 cm^−1^, a position typical of antiparallel β-sheet conformation; this feature is also evident in the spectra displayed in Figure 4, clearly showing that the peptide secondary structure is retained after adsorption on the HAP surface. The sharpness of the N-H stretching peak at 3270 cm^−1^ is a further proof of the peptide stereoregularity.

To furtherly check the SAP overlayer order and orientation on the HAP surface, NEXAFS investigations were carried out.

#### 3.2.2. NEXAFS Spectra

The SAP structure after adsorption on the HAP surfaces was also investigated by NEXAFS spectroscopy at the C and N K-edges. NEXAFS spectra of the investigated SAP deposited on titania were the object of previous studies [22,23]. The measured spectra for the HAP/SAP systems show the same features detected for TiO_2_/SAP systems, and peak assignment was made accordingly.

**Figure 5:**
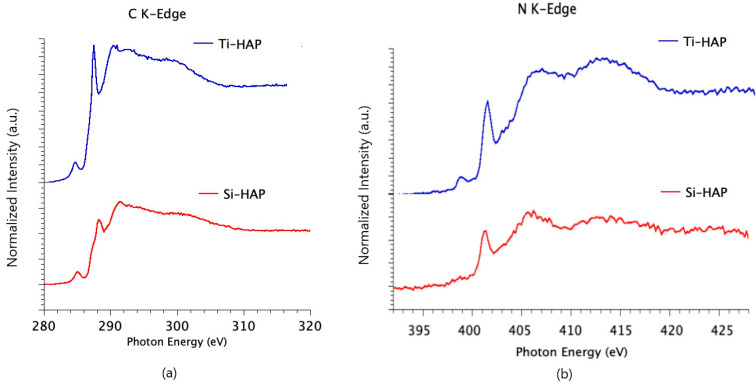
C K-edge (**a**) and N K-edge (**b**) NEXAFS spectra of Ti-HAP and Si-HAP after incubation with SAP solution at pH = 4, collected at magic incidence angle of 54.7°.

The C K-edge spectra of samples Ti-HAP and Si-HAP, after incubation with SAP solution at pH = 4, are shown in Figure 5a; according to XPS results, this pH seems to provide the best condition for obtaining thick SAP films on the HAP surface. The sharp feature at about 288.3 eV is related to a C1s→π* transition of C=O molecular orbital, and features at about 291.5 and 301 eV are associated to 1s→σ* transitions by the C–C and respectively C=O molecular groups. 

The N K-edge spectra of the same samples (reported in Figure 5b) show a sharp peak at 401.5 eV that can be assigned to N1s→π* transitions related to the peptide bonds, and two bands at 406 and 413 eV due to N1s→σ* _N-H_ and N1s→σ* _N-C_ resonances, respectively. 

**Figure 6:**
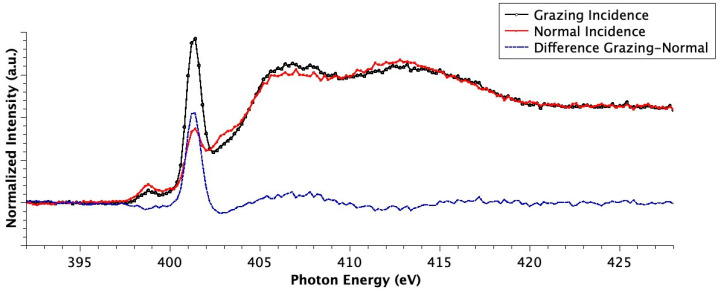
Angular dependent N K-edge NEXAFS spectra collected at Normal (90°, red line) and Grazing (20°, black line) incidence angles of the impinging X-ray beam on Ti-HAP after incubation with SAP solution at pH = 4. The difference (Grazing–Normal) spectrum, evidencing dichroic effects, is also shown (blue line).

All samples were also investigated by angular dependent NEXAFS spectroscopy. In order to determine the possible presence of a molecular preferential orientation, NEXAFS investigations at the N K-edges were performed by changing the incidence angle of the X-ray beam with respect to the sample surface from grazing (20°) to normal (90°). The dichroic effect for the N K-edge spectra collected on sample Ti-HAP is shown in Figure 6.

The angular dependence of the NEXAFS spectra of the SAP deposited on the HAP surface is unexpected, considering roughness of the HAP surface. Nevertheless, the effect shown in Figure 6 is evident. The significant difference in the intensities of the N1s→π* transitions detected under two different angles is caused by ordered arrangement of the peptide overlayer. This effect could be possibly related to the formation of a thick SAP overlayer (see measured film thickness in Table 4), having an ordered structure and a well-defined molecular orientation with respect to the substrate surface. 

Peptide self-assembling is expected to happen in water solution before adsorption of the assembled system on the HAP surface. We can make the hypothesis that rough samples, such as HAPs, having a higher active surface with respect to a flat one, could promote the adhesion of a higher amount of peptide that could get “entrapped” among the irregularities, thus forming a thicker layer. 

The dichroic effect evidenced for the N1s→π* transition related to the peptide bond can be used to determine the tilt angle between the π* orbital and the normal to the sample surface and subsequently the angle between the axis of the peptide backbone and the sample surface. We have calculated the ratio between the π* peak intensities at normal (90°) and grazing (20°) incidence, by peak fitting of the experimental data, and used it to calculate the average tilt angle, as reported in [22,23,41]. This allowed us to determine an angle of 77° between the peptide backbone and the sample surface for all the investigated samples; similar values were obtained for SAP immobilized on the TiO_2_ surface [22,23,31].

## 4. Discussion

The adsorption of an SAP peptide on the surface of doped HAP coatings, containing Mg, Ti or Si compounds incorporated in the HAP lattice, deposited on Ti6Al4V samples, was investigated at two different pH values of the mother solution, by XPS, NEXAFS and FTIR spectroscopy.

Experimental data show that SAP immobilization on both Ti-HAP and Si-HAP surfaces was successful. The peptide chemical structure is not perturbed by chemisorption, as proved by FTIR and XPS results. Incubation in peptide solution, on the other hand, produces a substantial reorganization of the outmost HAP substrate, inducing a migration of phosphate anions to the HAP surface. However, FTIR results prove that, below the top 50 Å of the surface, the structure of the HAP appears unaltered, even after treatment with acid SAP solutions. This phenomenon could be related to the quick formation of a relatively thick SAP overlayer that protects from dissolution the HAP coating underneath. 

SAP deposition is particularly successful for the TiO_2_ containing apatite, Ti-HAP, while a lower amount of adsorbed SAP is detected for Si-HAP, yielding evidence that TiO_2_ plays an important role in supporting peptide adhesion to the HAP surface. Moreover, the thickness of the peptide overlayer is also influenced by the pH value of the mother solution; thicker films are obtained in mildly acidic environment for the Ti-HAP sample, in analogy to results obtained for SAP adsorption on TiO_2_ [23]. The SAP adsorption on Si-HAP, on the other hand, is rather low at both pH values.

The secondary structure of the SAP adsorbed on the HAP surface is antiparallel β-sheet, as proved by FTIR investigation, resulting in being unperturbed by adsorption on HAP; in fact, SAPs tend to self-assemble in watery solution, and the assembled structure is immobilized on the substrate surface. Consequently, thick peptide overlayers immobilized on Ti-HAP, investigated by angle-dependent NEXAFS spectroscopy, unexpectedly show ordered structure and molecular orientation.

The approximate length of the SAP chain is estimated to be about 64 Å for a 16-units peptide in rigid β-sheet conformation, considering a 4 Å step for each amino acid, on the basis of average C–C (1.55 Å) and C–N (1.47 Å) bond lengths and of bond angles of 120°, as expected for sp^2^ hybridization. Taking into account a tilt angle of 77° between the peptide chain axis and the sample surface, the formation of a SAP monolayer on the HAP surface would result in an overlayer thickness of approximately 60 Å for Ti-HAP, and the measured value at pH = 4 is compatible with the formation of a complete monolayer of SAP, while for pH = 10, the measured film thickness is only slightly lower. Si-HAP, on the other hand, shows much lower thickness values for the SAP overlayer at both pH values, despite the same molecular orientation of the peptide backbone can be inferred from NEXAFS spectra. This is indicative of a submonolayer regime for the SAP deposited on Si-HAP; since the β-sheet conformation and the molecular order of the SAP are confirmed by FTIR and NEXAFS spectra, we can hypothesize the formation of islands of SAP on the Si-HAP surface.

## 5. Conclusions

SAP adsorption on the surface of HAP coatings enriched with, respectively, titanium or silicon, evidence the efficacy of TiO_2_ in supporting peptide adhesion to the HAP coating. The thickness of the peptide overlayer is in the monolayer regime for Ti-HAP, in the submonolayer regime for Si-HAP. The formation of an ordered SAP overlayer is related on the HAP surface to the SAP ability to self-assemble in aqueous solution before adsorption on the HAP surface.

## Figures and Tables

**Table 1:** RF power fed applied on used cathodes.

Sample	RF Powers Fed (W)			
	HAP Cathode	SiC Cathode	TiO_2_ Cathode	MgO Cathode
Ti-HAP	50	-	25	50
Si-HAP	50	15	-	50

**Table 2:** Photoemission peaks of the HAP samples.

Sample	BE (eV)						
	Ca2p_3/2_	P2p	Si2p	Ti2p_3/2_	Mg2p	O1s(0)	O1s (1)
Ti-HAP	347.5	133.6		458.8	50.3	530.4	531.6
Si-HAP	347.5	132.4	102.6		50.9		531.9

**Table 3:** Measured atomic ratios of investigated samples.

Sample	P/Ca	Si/Ca	Ti/Ca	Mg/Ca	O/Ca	C/Ca	O_0_/O_1_
Ti-HAP	0.57		0.39	0.34	3.8	3.2	0.54
Si-HAP	0.40	0.54		0.15	2.48	3.6	

**Table 4:** Measured atomic ratios and overlayer thickness (Å) after samples incubation in SAP solutions at pH values of 4 and 10.

Sample	pH	C/Ca	N/Ca	O/Ca	P/Ca	Ti/Ca	C_2_/C_1_	C_3_/C_1_	N_2_/N_1_	O_0_/O_1_	O_2_/O_1_	Å
Ti-HAP	4	35	4.3	17	2.1	0.	0.5	0.3	0.14	0.16	0.16	59
	10	15	1.1	13	1.2	1.5	0.6	0.5	0.18	0.4	0.3	43
	**pH**	**C/Ca**	**N/Ca**	**O/Ca**	**P/Ca**	**Si/Ca**	**C_2_/C_1_**	**C_3_/C_1_**			**O_2_/O_1_**	**Å**
Si-HAP	4	10	0.23	5.0	0.9	0.2	0.4	0.25			0.18	9
	10	8	0.26	6.6	0.7	0.9	0.3	0.2	0.1		0.11	17
